# Designing Antibacterial-Based Quaternary Ammonium Coatings (Surfaces) or Films for Biomedical Applications: Recent Advances

**DOI:** 10.3390/ijms252212264

**Published:** 2024-11-15

**Authors:** Georgia C. Lainioti, Denisa Druvari

**Affiliations:** 1 Department of Food Science & Technology, University of Patras, GR-30100 Agrinio, Greece; 2 Department of Chemistry, University of Patras, GR-26504 Patras, Greece; druvari@upatras.gr

**Keywords:** antibacterial coatings, quaternary ammonium compounds, control release, mechanism of action, contact killing, dual-functional coatings

## Abstract

Antibacterial coatings based on quaternary ammonium compounds (QACs) have been widely investigated in controlled release applications. Quaternary ammonium compounds are low-cost and easily accessible disinfectants that have been extensively used, especially after the COVID-19 outbreak. There has been a growing interest in developing a clearer understanding of various aspects that need to be taken into account for the design of quaternary ammonium compounds to be used in the biomedical field. In this contribution, we outline the mechanism of action of those materials as well as the key design parameters associated with their structure and antibacterial activity. Moreover, emphasis has been placed on the type of antibacterial coatings based on QACs and their applications in the biomedical field. A brief outlook on future research guidelines for the development of dual-function antibacterial coatings is also discussed.

## 1. Introduction

Microbial infection poses a significant global health challenge, impacting human wellbeing on a worldwide scale. Pathogenic microorganisms, such as bacteria, viruses and fungi, are a cause for concern in hospitals and other healthcare environments. They have a detrimental impact on the proper functioning of medical devices, pharmaceuticals, surgical instruments, bone cements and dental restorations [1,2,3].

The number of resistant bacterial strains is constantly growing and is a matter of great concern for the scientific community; if it remains unaddressed, antimicrobial resistance-related deaths could overtake those caused by cancer by 2050 [4,5,6].

The global focus on contamination through high-touch surfaces has been heightened by the SARS-CoV-2 (COVID-19) pandemic, which began in 2020 [7]. The increased use of disinfectants has resulted in an elevated risk of bacterial resistance development, which could potentially diminish the efficacy of biocidal treatments in the future [8,9]. As a result, there is high interest in developing surfaces and coatings that can effectively reduce the presence of active viral pathogens in order to be used in applications, including in health care services, public transport, schools and a variety of business contexts. The aim is to minimize human exposure and reduce the spread of infectious pathogens.

Within this context, the field of antibacterial coatings has gained considerable attention in research. Advancements in material design, processing and surface modification techniques have fueled the creation of surfaces capable of inhibiting bacterial adhesion, growth and biofilm formation [10]. Consequently, these coatings hold the potential to reduce the risks of infections associated with biomedical devices.

It is important to note that antibacterial polymeric coatings can not only be used independently, but they can also attach to a surface with no loss of their biological effectiveness. The last approach enables the development of coatings that inactivate bacteria without the need for additional biocides to be released or that reinforce the antimicrobial effectiveness of such biocides by working together in an additive or synergistic manner.

The interface between a material’s surface and external elements is vital and influenced by a range of properties, including thermodynamic, mechanical, chemical and biological characteristics. Consequently, altering surface properties and interactions through surface modification presents a practical approach while preserving the material’s structural integrity [11,12]. Various techniques exist for surface modification, including physical, thermal and chemical methods, such as surface treatments and coatings [13]. Surface coating refers to the application of a thin layer of coating material onto a larger material. This can be achieved through different techniques, such as thermal spraying, electrospraying, vapor deposition or the use of polymeric coating layers [14]. Between these methods, polymer coating represents a simple and cost-effective method that has been extensively researched and applied to various materials. Its aim is to enhance or modify properties related to corrosion, adhesion, material strength, abrasion, energy efficiency, biocompatibility and usability [15].

**Figure 1:**
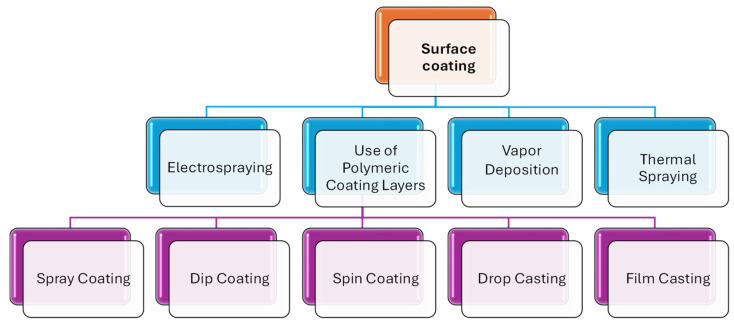
Schematic representation showing the surface coating preparation process.

Polymer coating techniques include spray coating, dip coating, spin coating, drop casting and film casting [16]. These techniques can be used to create a protective layer on a solid surface and impart desired surface properties, such as scratch, water and corrosion resistance. They are widely applied in industries such as painting, construction, automotive manufacturing and biomedical device manufacturing. Figure 1 depicts a schematic representation of the surface coating preparation process.

The antimicrobial compounds that are most commonly used in contact-killing coatings involve quaternary ammonium compounds (QACs), antimicrobial peptides (AMPs), silver nanoparticles (AgNPs), chitosan and antimicrobial enzymes (AMEs) [17]. Quaternary ammonium group-containing polymers have emerged as extensively researched macromolecular antimicrobial agents. These polymers are frequently utilized as antimicrobial coatings due to their biocidal properties, attributed to the presence of positively charged ammonium moieties [18].

QACs gained attention as effective bactericides in the 1930s [19]. QACs consist of at least one positively charged nitrogen atom (N^+^), which can originate from the N^+^ in the aromatic ring (imidazole, quinoline, isoquinoline, pyridine) or the N^+^ on the straight chain. The N^+^ forms covalent bonds with four carbon atoms, with one of the chains acting as a substituent, and the overall length ranges from C8 to C18. Their enhanced water solubility can be attributed to the hydrophilic portion, while the accompanying anion is typically chloride (Cl^−^) or bromide (Br^−^).

The QAC has a general formula of N^+^R_1_R_2_R_3_R_4_X^−^, wherein R may denote a hydrogen atom, a simple alkyl group or an alkyl group bearing additional functional groups, while X represents an anion. Long-chain QACs containing 8–18 carbon atoms generally exhibit potent antibacterial activity. Notable examples within this category include benzalkonium chloride, stearalkonium chloride and cetrimonium chloride [20]. These compounds demonstrate effectiveness against both Gram-positive and Gram-negative bacteria, fungi and certain types of viruses. The antimicrobial efficacy of QACs is influenced by various factors, such as the length of the alkyl chain, the presence of halogenated groups and the number of cationic ammonium groups in the molecule.

The antimicrobial mechanism involves initial electrostatic interactions between the positively charged N^+^ atom and the negatively charged cell membrane of the microorganism, followed by intercalation and subsequent disruption [21].

The distinctive structure of QACs makes them excellent candidates for use in clinical settings, agriculture, food industry, household, construction and other fields [22,23]. Examples of their applications range from water treatment [24], oil production [25], textile production [26], preventing mold in the food industry [27], protection of wood and building materials [28,29], sterilizing surgical and medical equipment [30], egg micro seal protection [31] and healthcare antiseptics [32].

However, adverse effects may occur when concentrations of QACs approach levels of concern, including severe and chronic toxicity to susceptible aquatic organisms, animals and humans. QACs become lethal to aquatic organisms by generating oxidative stress, disrupting membrane integrity and creating molecular defects due to the induction of apoptosis and endoplasmic reticulum stress [33]. It has been reported that QACs enhance the release of cyanotoxins and therefore may cause damage to various organisms, such as freshwater crustaceans, fish and algae. QAC toxicity is quite variable depending on the species and algae may be highly sensitive because their cell walls bear a generally negative charge [34]. Mixtures act synergistically or antagonistically depending on the concentration, whereas individual QACs, especially those with longer carbon chains, have higher toxicity than their shorter-chain analogues.

Animal model studies conducted on QACs, including *n*-alkyl dimethyl benzyl ammonium chloride (ADBAC) and didecyl dimethyl ammonium chloride (DADMAC C10), showed a potential decrease in fertility and problems in fetal development [35]. Mice with high-dose exposure recorded lower pregnancies and fewer offspring. In the complicated reproductive activities, both sexes were implicated, including low ovulation rates among females as well as low sperm concentration and motility among males. In mice, neural tube defects and reproductive defects have been recorded even at environmentally relevant exposures. Even in view of this finding, the high-dose studies have been criticized, and human exposure limits from a lack of data restrict relevance to human health. Further research is necessary for understanding QAC impacts on human fertility and development.

Human exposure to QACs primarily occurs through inhalation, skin contact and ingestion, the latter usually being more hazardous due to concentrated QACs. Highly concentrated QACs induce disfunction in mitochondria, leading to cell death and a predisposition towards asthma in healthcare workers [36]. During the COVID-19 pandemic, studies revealed that QACs were found in many individuals’ blood due to bioaccumulation, inflammation and disturbance in cholesterol metabolism [37]. More specifically, benzalkonium chloride related to the alterations in gene expression influencing cholesterol synthesis [38]. While toxicity due to QACs was tested individually, additive effects might occur, and further research would be necessary for safer alternatives in disinfection with a view to minimizing health risks.

This review focuses on the most recent advances in antimicrobial coatings based on Quaternary Ammonium Coatings intended for Biomedical Applications and our perception on the most prominent method applied in each case. The article introduces an overview of the issue of infection and its impact on healthcare. The following section outlines the mechanism of action of QACs and the relationship between the structure and antibacterial activity. The following part of the article discusses the four distinct classifications of antibacterial coatings and materials: bacteria-repelling, contact-killing, releasing and responsive. These classifications have been developed by our team and other researchers over the years. The article presents specific examples of antibacterial materials for each category and provides a discussion of the pros and cons related to each approach.

In our view, the potential future application of antibacterial coatings based on QACs in drug delivery systems or for the surfaces of medical devices (e.g., implants, prostheses, stents, surgical sutures, etc.) may reduce the use of generally familiar biocides, particularly overused antibiotics.

In certain instances, the coating process involves applying electrospinning, dip-coating and polyelectrolyte complex formation onto a polymeric support. However, in other cases, modified surfaces are achieved through the direct covalent linkage of quaternary ammonium salts onto the polymeric support.

## 2. Mechanism of Action of QACs

There exists a variety of viewpoints regarding the antibacterial mechanism of QACs [39]. However, the membrane-active mechanism is the most prevailing perspective to the best of our knowledge [40,41]. The bacterial membrane plays a vital role in all stages of bacterial life, housing nearly a third of the proteins involved in bacterial metabolism as well as maintaining cellular balance. Thus, imitating AMPs [42], QACs focus on the bacterial membrane as the prime target, due to its considerable importance.

**Figure 2:**
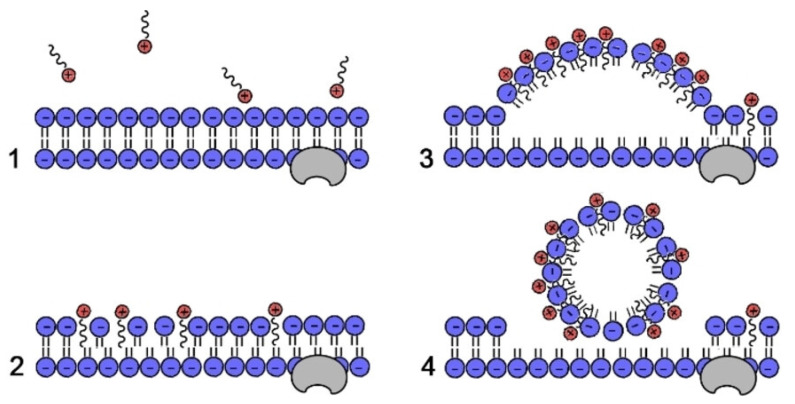
General mechanism of action of quaternary ammonium salts (QAS), where the phospholipid membranes are depicted in blue and the QAS are shown in red. (1) Adherence to the bacteria’s surface through electrostatic forces; (2) Penetration of the cell membrane and attachment to it; (3) Reaction with cytoplasmic membrane to cause disorder; and (4) Membrane damage (Reproduced from Ref. [53], with the permission of Wiley).

It is generally acknowledged that the process of bacterial membrane disruption can be divided into four stages, as shown in Figure 2: (1) adherence to the bacteria’s surface through electrostatic forces; (2) penetration of the cell membrane and attachment to it; (3) reaction with cytoplasmic membrane to cause disorder; and (4) membrane damage, leading to bacterial death. These four stages are driven by a series of molecular interactions, including electrostatic, hydrophobic and ionic forces, which together facilitate the disruption of the bacterial membrane and contribute to the eventual leakage of intracellular substances, leading to bacterial death. Each of these interactions play a critical role in the effectiveness of quaternary ammonium compounds (QACs) as antimicrobial agents, starting with their electrostatic attraction to the bacterial surface.

Electrostatic Interactions: This first step in the interaction between QACs and the bacterial cell wall is primarily driven by electrostatic forces. The surface structure of bacterial cell wall, especially Gram-negative bacteria, bear a predominantly negative charge, as they consist of phospholipids and lipopolysaccharides [43]. QACs possessing a cationic nature are hence attracted to these negatively charged constituents. This electrostatic attraction enables QACs to be adsorbed into the bacterial surface, an important precursory step before their action against microbes. For example, Wang et al. have demonstrated that cationic quaternary ammonium groups interact with the anionic phosphoryl groups in the bacterial membrane, leading to changes in membrane permeability and eventually to cell lysis [44].

Hydrophobic Interactions: In addition to the primary electrostatic interactions, hydrophobic forces play a significant role in the efficacy of QACs. The presence of long alkyl chains in QACs enhances their hydrophobicity, allowing them to penetrate the membrane lipid bilayer more effectively [45]. As described by Mokeem et al. [46] and Hoque et al. [47], hydrophobic interactions aid in the initial binding of QACs to the bacterial membrane, inducing a destabilization within the structure of the membrane, which then becomes permeable.

Interaction with Membrane Stabilizing Cations: QACs also interact with membrane-stabilizing cations, such as magnesium (Mg^2+^) and calcium (Ca^2+^), which are crucial for maintaining the integrity of bacterial membranes. By displacing these cations, QACs destabilize the membrane structure, leading to increased permeability and cell lysis [48]. This interaction highlights the importance of ionic balance in bacterial survival and the effectiveness of QACs as antimicrobial agents.

Cytoplasmic Leakage and Cell Death: Ultimately, through these interactions, QACs are reported to cause serious damage to the cytoplasmic membrane, leading to the leakage of key cellular components, such as potassium ions, DNA and RNA [49,50], which is a hallmark of bacterial cell death. This mechanism is quite effective against Gram-positive and Gram-negative bacteria, except that Gram-positive bacteria are more susceptible because their cell wall composition is simpler [51].

Consequently, it is proposed that QASs primarily exert their bactericidal effect through the hydrophobic action of long carbon chains and the electrostatic attachment of cations to negatively charged bacterial surfaces [52,53].

In addition, polymeric materials bearing QACs function in a similar way as described above, through membrane disruption. For instance, polyethylenimine is a well-studied antimicrobial synthetic polymer which leads to the disruption of the cell membrane due to electrostatic interactions between the cationic groups of the polymer and the negatively charged outer cell membrane of bacteria [54]. Chitosan is an example of a natural antibacterial polymer that has been studied thoroughly in its quaternized form. It attaches to the bacterial surface membrane through electrostatic forces, which causes membrane stripping, enhancing its permeability and leading to an irregular distribution of cytoplasm [55].

## 3. Association Between Structure and Antibacterial Activity

There is a significant gap in our understanding of the specific combination of molecular characteristics of membrane-active compounds that dictate how they interact with the lipid membranes of various species. This knowledge gap presents noteworthy impediments to the rational design of compounds that would interact with membranes in a targeted manner. Many antimicrobial compounds are effective against the membranes of a range of species, including both prokaryotic and eukaryotic cells, which can lead to significantly harmful effects by disrupting the host cell’s membrane [56]. Consequently, a primary objective in the development of next-generation antimicrobials is to minimize these side effects and achieve selectivity for specific pathogenic bacteria.

Given that the complex nature of these molecular interactions must be carefully balanced, combination techniques are important for the exploration of structure–activity relationships compared to traditional step-by-step scientific approaches. While high-throughput screening of AMP structures has made progress, research into synthetic amphiphiles, like QACs, has typically focused on twin-tail “gemini” compounds, which consist of two polar ammonium head-groups and two hydrophobic aliphatic tails [57,58]. Octenidine (OCT) is an example of a highly effective broad-spectrum gemini QAC that is extensively used as an antiseptic [59].

Some studies suggest that “multi-QACs” with more than two charged headgroups and multiple tails may outperform conventional gemini QACs [60,61], but the reasons for why increasing structural complexity affects antimicrobial activity are not well-explored. There is also a growing interest in developing systems with highly charged headgroups, as both QACs [62] and AMPs containing a greater number of charged components (more than 10) have been found to be more challenging for bacteria to acquire resistance against [63,64].

In general, the antimicrobial effectiveness of QACs depends on various factors, with the alkyl chain length, the type of counterion and the number of cationic ammonium groups within the molecule being the most explored. They can be categorized into mono-, bis- and multi-QACs depending on the number of quaternary nitrogen sites and the resulting overall charge of the compound. Herein, the latest research on the structure-related factors affecting the antimicrobial efficacy of QACs is presented.

### 3.1. Type of Counterion

Quaternary ammonium compounds exhibit an amphiphilic structure characterized by a hydrophilic (lipophobic) head and a hydrophobic (lipophilic) tail, which interact with cell membrane lipids. The effectiveness of QACs is boosted by counterions, especially reactive ones that can oxidize proteins or lipids. Particularly potent are compounds containing chlorine, bromide or iron atoms [65,66,67]. It is suggested that when the anion is strongly bound to the N^+^ atom, it reduces the adsorption of positively charged N^+^ to the cell membrane and the ion exchange with Ca^2+^ and Mg^2+^ on the membrane surface, leading to reduced bactericidal activity. However, there is a growing demand for new types of counterions that align with the criteria for safe disinfection agents and the principles of “green chemistry”. Notably, there is interest in counterions like lactates, formates and acetates.

In a recent work, QACs derivatives containing innovative counterions were synthesized through a process involving the quaternization of a suitable tertiary amine derivative with dimethyl carbonate, followed by a reaction with a stronger acid, such as acetic, lactic or formic acid, compared to carbonic acid [68]. Notably, the nature of the counterion had no significant effect on antimicrobial activity against planktonic forms. However, mono-QACs with acetate or bromide as counterion were somewhat more effective in eliminating the biofilm of all tested strains. Additionally, bromide as the counterion likely increased the mutagenic properties of compounds containing 12 and 16 carbon atoms in the chain.

### 3.2. Mono-QACs

In general, it is widely held that the optimal impact of QACs is observed within a specific range of alkyl chain lengths. Specifically, QACs with alkyl chains of 10–16 carbons are particularly potent against microbes, mainly attributed to the formation of highly effective bipolar dimers [69]. Conversely, having alkyl chains longer than C18 is not advisable, primarily due to the limited solubility of such agents. Long alkyl chains, such as C16, appear to exhibit greater effectiveness against Gram-negative bacteria, while shorter chain lengths have a more favorable efficacy against Gram-positive bacteria and yeasts [70]. Notably, double-chained compounds like dialkyl dimethyl ammonium compounds (DADMACs) have superior activity against Gram-positive bacteria compared to Benzalkonium chlorides (BACs) [71].

Bazina et al. have reported on a series of new QACs based on quinuclidine-3-ol. Notably, the compound *N*-tetradecyl-3-hydroxyquinuclidinium bromide demonstrated high efficacy against Gram-positive bacteria such as *S. aureus*, MRSA, *S. pyogenes*, *E. faecalis* and *L. monocytogenes*, with MIC values ranging from 0.06 to 3.9 μg/mL [72].

Zhang et al. have synthesized novel β-pinene QACs and assessed their antibacterial activity. Notably, a bi-substituted derivative (bis-hydronopyl quaternary ammonium compound) displayed broad-spectrum activity against *E. coli*, *S. aureus* and *P. aeruginosa,* with MIC values between 0.625 to 2.5 μg/mL. It is noteworthy that halide ions significantly influenced the activity, with Cl ion being more effective than I ion [73].

**Figure 3:**
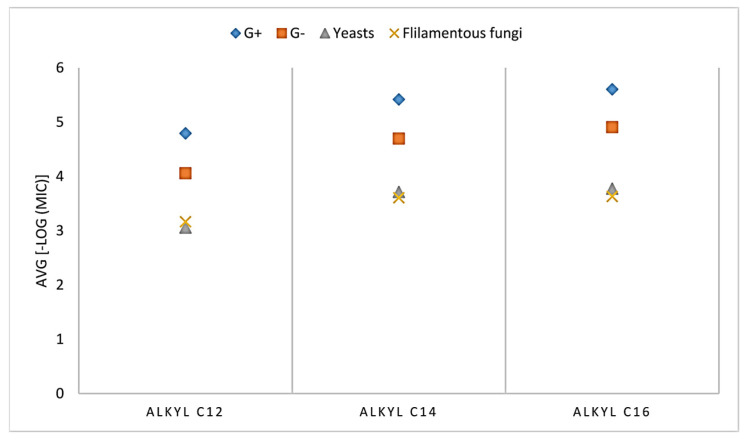
The impact of alkyl chain length on the effectiveness against bacteria, yeasts and fungi. (Reproduced from Ref. [73], with the permission of Elsevier).

In a another study, Soukup et al. prepared 43 novel *N*-alkyl monoquaternary ammonium salts and 7 *N*,*N*-dialkyl monoquaternary ammonium salts bearing different alkyl chains: containing (i) a heterocyclic core with an attached alkyl chain (C12, C14, C16) or (ii) a heterocyclic ring with a shorter methylene tether (C8, C10) [74]. It was demonstrated that there is a variation in the effects of individual QACs against various types of microbes involving Gram-positive and Gram-negative bacteria, filamentous fungi, anaerobic spore-forming *Cl. difficile*, yeasts and enveloped Varicella zoster virus (VZV). As can be seen in Figure 3, the results indicated that antibacterial activity is enhanced with a larger molecule, more lipophilic and less polar.

### 3.3. Multi-QACs

Recent advancements in this area have demonstrated that bis- or multi-cationic compounds exhibit superior antimicrobial efficacy compared to mono-QACs [75].

Kontos et al. prepared some DABCO-based (1,4-diazabicyclo[2.2.2]octane) bis-cationic QACs, which exhibited strong effectiveness against *S. aureus*, *E. coli*, *E. faecalis*, *P. aeruginosa* and MRSA, with a maximum MIC of 0.25 μM (in the case of dodecyl side chain substituted, DABCO-12,12) [76]. Shtyrlin et al. [77] reported on the synthesis of a series of 108 novel bis-cationic QACs derived from pyridoxine. Among those, 13 compounds demonstrated antibacterial and antifungal activity with MIC values ranging from 0.25 to 16 µg/mL, comparable to, or surpassing those of, miramistin, benzalkonium chloride and chlorhexidine. Notably, a strong relationship concerning antibacterial activity and lipophilicity was observed, with the dodecyl substituted analogue showing the highest activity against *S. epidermidis*, *S. aureus*, *E. coli*, *B. subtilis*, *K. pneumoniae* and *P. aeruginosa* with MIC values ranging from 1 to 32 μg/mL. Leitgeb et al. [78] synthesized 36 new bis-QACs featuring different two-carbon linker geometries based on alkane, alkene and alkyne cores, aiming to evaluate how the geometry of QACs influences their antimicrobial activity. Some dialkyl pyridinium bis-QACs showed promising activity against the tested microorganisms, with the superior MIC of 1 μM against MRSA in the case of the dodecyl-substituted compound. Guo et al. [79] synthesized bis-cationic QACs derived from honokiol by replicating cationic antimicrobial peptides’ chemical structures and antibacterial properties, which demonstrated potential efficacy toward clinical MRSA isolates with MIC values of 0.5–1 μg/mL.

**Figure 4:**
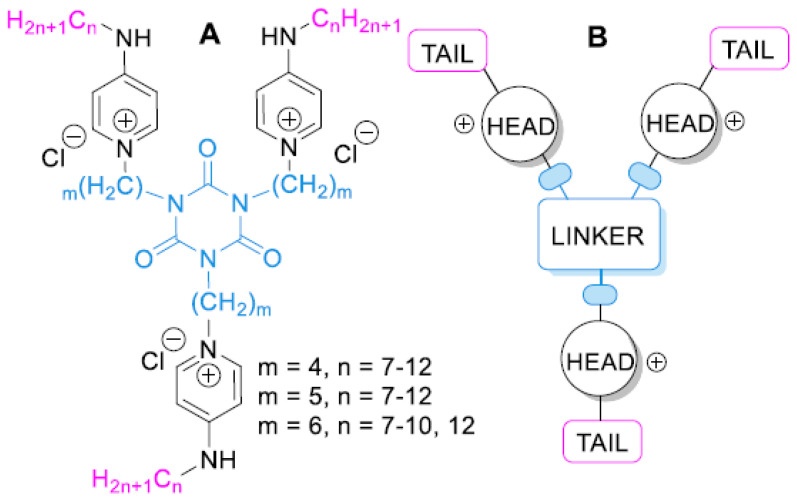
(**A**) General method of the recently developed trimeric QACs. (**B**) Conventional names of structural elements of the QACs. (Reproduced from Ref. [83], with the permission of ACS Publications).

The group of Vereshchagin has proposed multiple ways to obtain antimicrobial multi-QACs lately. They synthesized new tetrakis–quaternary ammonium compounds based on pentaerythritol and 3-hydroxypyridine [80], novel cyanuric acid-tethered tris-pyridinium derivatives [81], whereas they showed that novel pyridinium bis-QACs with aromatic spacers exhibited a wider range of antibacterial activity compared to the commonly used antiseptics of benzalkonium chloride and cetylpyridinium chloride [82]. In their latest work, they studied the newly developed tris-QACs, shown in Figure 4, which were demonstrated to exhibit strong biocidal activity with no detected tolerance in *S. aureus* and significant potential to be used against Gram-negative pathogens as well as the yeast-like fungus *Candida albicans* [83].

### 3.4. Gemini QACs

The existence of two alkyl chains and two ammonium cations within the molecule makes gemini QACs significantly more effective biocides compared to their monomeric counterparts [84,85,86,87]. The MIC values of gemini QACs are typically several orders of magnitude lower than those of similar QACs. GSs exhibit a broad spectrum of activity against bacteria.

Gram-negative bacteria are less susceptible to microbiocides than Gram-positive bacteria due to the more intricate structure of their cell wall. Consequently, microorganisms show sensitivity to gemini surfactants (GSs) in the following order: Gram-positive bacteria > fungi > Gram-negative bacteria [88].

Gemini QACs with various counterions (bromide, hydrogen chloride, methylcarbonate, acetate and lactate), different chain lengths (C12, C14 and C16) and a methylene linker (3xCH_2_) were recently studied [89]. The results showed that dihydrochlorides and dibromides with 12-carbon hydrophobic chains exhibited the highest activity against planktonic forms and fungi (*C. albicans*), while the gemini compounds containing 16-carbon alkyl chains proved to be the most effective in inhibiting biofilm formation.

**Figure 5:**
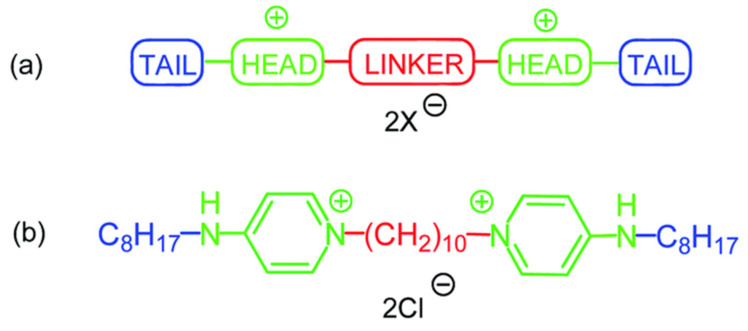
(**a**) General structure of cationic gemini surfactants, (**b**) structure of octenidine (Reproduced from Ref. [80], with the permission of Royal Society of Chemistry).

Eighteen new gemini quaternary ammonium compounds were synthesized by the group of Vereshchagin to investigate the impact of the linker type, the aliphatic chain length and their relative positions on antibacterial and antifungal activity, as shown in Figure 5 [80]. Overall, for bis-QACs that contain a biphenyl linker, the order of antibacterial activity decrease is: meta- > para- > ortho-. In contrast, the activity decrease for those with an oxybiphenyl linker, follows the order: para- > meta- > ortho-. The gemini amphiphile 4OB4POB-9,Br, which has an oxydiphenyl linker and a C_9_H_19_ alkyl substituent, was identified as significantly superior to the widely used commercial antiseptics.

In another recent study, 16 novel Octenidine (OCT) derivatives were synthesized and tested as a new class of gemini QACs biocidals [90]. It was observed that the length of the alkyl side chain plays a critical role in antimicrobial activity against Gram-positive strains, while the structure of the linker chain does not appear to affect the activity. Several highly promising compounds from this study were identified; however, compound 1,1′-(2-oxapropane-1,3-diyl)-bis[4-(decylamino)pyridin-1-ium] dichloride demonstrated excellent results against both Gram-positive and Gram-negative bacteria, nearly all types of fungi and HSV1, while also exhibiting lower cytotoxicity than OCT.

## 4. Antibacterial Coatings Based on QACs

**Figure 6:**
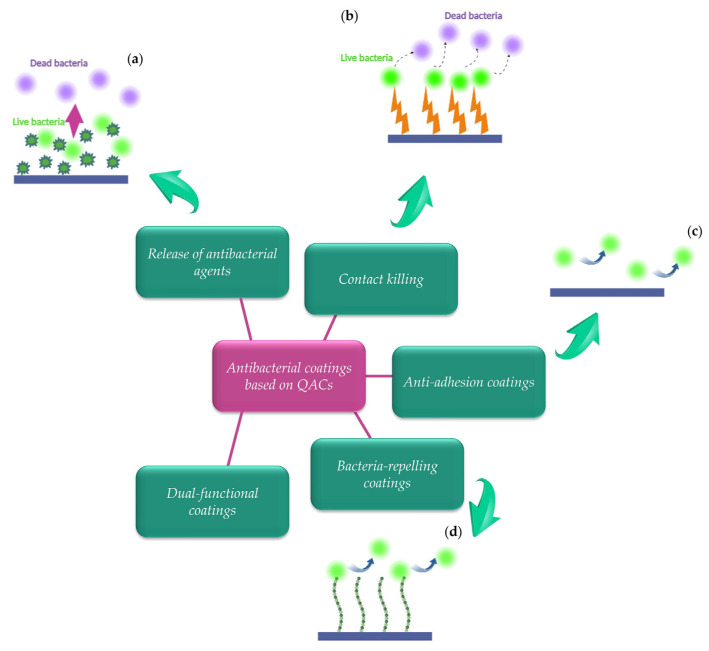
Illustrations of (**a**) release of antibacterial agents, (**b**) contact-killing, (**c**) Anti-adhesion and (**d**) Bacteria-repelling approaches of antibacterial coatings.

Due to the increasing demand for finding alternative solutions to prevent antibiotic use, the development of potent antibacterial coatings has arisen as a considerable challenge in the area of biomaterial design. Consequently, there has been a notable rise in research activity concerning antibacterial surfaces or coatings, motivated by the pressing need to find practical alternatives [91]. Form an extensive review in literature there are specific approaches that are used for the enhancement of antibacterial surfaces. These strategies, each presenting its own set of benefits and drawbacks, include controlled release of antibacterial agents, contact killing mechanisms and techniques that prevent bacterial adhesion or repel bacteria from the surface. Figure 6 depicts the types of antibacterial approaches employed by antibacterial surfaces or coatings.

These approaches may be used either independently or in combination, in order to achieve synergistic effects, for the enhancement of the antimicrobial characteristics of a surface or coating [92]. Nevertheless, irrespective of the selected approach and surface or coating design, specific key characteristics are required for antibacterial surfaces, such as biocompatibility, durability, efficacy against target pathogens and the ability to maintain stability over time [92].

The antimicrobial activity of polymers with cationic groups, such as quaternary ammonium and phosphonium salts, relies significantly on the positively charged groups present in their structures, which play a vital role in attracting and disrupting microbial cell membranes through ionic interactions. This leads to leakage of intracellular components and ultimately causes cell lysis [93]. Moreover, the electrostatic interaction between cationic groups of polymers and the anionic moieties of extracellular polymers and bacterial cell walls enables the precise delivery of antimicrobial agents to the biofilm [94]. Cationic polymers offer several additional advantages for the advancement of antimicrobial coatings, i.e., chemical stability, ease of processing, a wide range of antimicrobial activity and an extended duration of effectiveness [95].

Current coatings designed to combat primarily bacterial fouling as well as contamination employ two approaches: the antibacterial, where bacteria are neutralized, and the antifouling, where bacteria are prevented from adhering. Within this context, below we present the mechanisms of action for the quaternary ammonium-based surfaces or coatings which are based on release inactivation, contact inactivation and repelling. Special attention is given to dual-functional coatings, which combine various mechanisms that act synergistically in order to provide multiple lines of defense against bacteria. Moreover, intelligent antibacterial coatings are also presented since they possess the capacity to respond dynamically to varying conditions and bacterial challenges, offering a higher level of efficiency and precision in their antibacterial actions. By incorporating intelligent features, such as responsive materials or controlled release mechanisms, these coatings can adapt to the presence of bacteria, targeting them more effectively while minimizing the risk of resistance development.

### 4.1. Release of Antibacterial Agents

Coatings containing releasable antibacterial agents are infused with antibacterial substances (such as nanoparticles, antibiotics and metal ions as well as their oxides) within the coating on the surface layer of the implant. These agents are then released in a controlled manner. These coatings may release bioactive substances at clinically effective concentrations, eradicating bacteria both near the surface and at a distance. Nevertheless, once the biocidal agent is completely used up within a certain time period, the antimicrobial properties of the surface are lost. Therefore, the optimal features of microbicide-releasing surfaces include a slow leaching or gradual releasing of the antimicrobial agent to extend antimicrobial duration before re-coating and the use of low levels of toxic microbicides for an effective microbial death [96]. To ensure a sustained and controlled release of agents for effective bacterial targeting, integrating releasable antimicrobial coatings with nanocomposites presents a promising and innovative approach. This integration can introduce additional functionalities, thereby enhancing the overall antibacterial performance.

Yu’s group [97] developed rough-surfaced mesoporous silica nanoparticles (MSNs) containing benzalkonium chloride (BAC), referred to as RNPs-BAC, using a soft template method. During synthesis, conventional spherical BAC-containing micelles were converted into rod-like micelles with the aid of sodium trifluoroacetate (FC2) in order to enable co-assembly with silica components, resulting in the formation of nanoparticles with an uneven surface texture. These rough nanoparticles exhibited enhanced bacterial adhesion and facilitated the localized release of BAC directly onto the bacterial surface. BAC encapsulation within the nanoparticles ensured a robust coating on the substrates, preventing rapid leaching and offering long-lasting antibacterial protection. Moreover, the in-situ assembly of BAC molecules that can be easily cleaned into nanoparticle-based formulations allowed for straightforward antibacterial film on a range of substrates, enhancing water resistance and durability. This broadens the potential applications of BAC, making it suitable for fabricating pathogen-killing masks, wound dressings and medical devices.

Stewart et al. [98] showed that, due to their micellar properties, QACs can serve as effective templates for the self-assembly of antimicrobial QAC/silica nanocomposites. Within this context, they demonstrated the co-assembly of an antimicrobial drug, named octenidine dihydrochloride (OCT), with silica, resulting in the formation of highly loaded OCT-silica nanocomposite spheres with 500 nm diameter. These nanocomposite spheres achieved a drug loading of 35% by weight. Notably, the drug release from these spheres exhibited significantly prolonged release compared to conventional OCT-loaded mesoporous silica. This innovative concept can be extended to a wide range of self-assembling drugs, providing the medical field with a valuable tool for bottom-up synthesis of drug-loaded inorganic nanomaterials.

In another work, Tan and co-workers [99] reported a promising nanocomposite material achieved through the non-covalent modification of reduced graphene oxide (rGO) with QACs (quaternary ammonium compounds). To regulate the long-term antibacterial efficacy of quaternary ammonium salts, the self-assembly of dodecyl dimethyl benzyl ammonium chloride (rGO-1227) and rGO-bromohexadecyl pyridine (rGO-CPB) was achieved on the surfaces of reduced graphene oxide (rGO) using π-π interactions. Both rGO-CPB and rGO-1227 demonstrated diminished cytotoxicity compared to the pure antimicrobial agents, while retaining robust antimicrobial properties. However, the nanocomposite rGO-CPB displayed a delayed release over 60 days, enabling the prolonged storage of CPB for an extended bactericidal effect.

In another study [100], a range of quaternized triblock copolymers, referred to as QP-b-PCL-b-QPs, were prepared and self-organized into reverse micelles (RMs) using tetrahydrofuran. Biocompatible poly(ε-caprolactone) (PCL) blocks were contained in the shell of RMs, ensuring biocompatibility and responsiveness, whereas biocidal quaternary blocks were in the core, conferring antibacterial activity. When bacterial lipase is present, the biodegradable PCL blocks undergo hydrolysis, leading to the controlled release of quaternary biocidal agents (QBAs), resulting in self-sterilization. These RMs may easily be incorporated into commercial gelatin sponge (GS) to create RM2-coated GS, showing strong antibacterial action upon exposure to lipase.

### 4.2. Contact Killing

Unlike antibacterial releasing coatings, the contact killing approach involves coatings that contain antimicrobial agents covalently bound to the material’s surface. These agents aim to kill microorganisms or inhibit their growth by penetrating the cell wall [10,101], resulting in bacterial death through direct contact with the cellular membrane. Because of the net negative surface charge that bacteria usually exhibit [102], contact killing coatings are most efficient when utilizing cationic compounds, such as quaternary ammonium compounds, antimicrobial cationic peptides and chitosan or enzymes like lysozyme and other proteases.

Ao et al. [103] recently introduced a combination coating for orthopedic implants based on hydroxypropyltrimethylammonium chloride chitosan (HACC). The coating employs both contact killing and release killing antibacterial strategies to achieve its antibacterial action. The researchers demonstrated that this multilayer coating effectively inhibits the formation of biofilms caused by various bacterial strains, comprising of clinical isolates of methicillin-resistant *Staphylococcus epidermidis* (MRSE 287) and methicillin-resistant *Staphylococcus aureus* (MRSA, ATCC 43300).

Zhao et al. [104] prepared monolayer bactericidal films using QAS-PNIPAM microgels. Bacterial experiments conducted on these films demonstrated an exceptionally high bactericidal efficiency, nearing 100%. Moreover, the particle film exhibited robustness and superior ability for film formation on various material surfaces, suggesting its potential as a multi-functional coating material for antibacterial purposes. Through systematic experimentation, it was observed that the decisive factor in bacteria killing was the chemical composition of the microgels rather than their topological structure. Remarkably, the QAS-PNIPAM microgels were also able to form films on a wide range of substrates, including metal materials, inorganic non-metallic materials, as well as plastics and elastomers. On these diverse substrates, the QAS-PNIPAM microgel films maintained high bactericidal activity. Thus, the QAS-PNIPAM microgels exhibited exceptional universality in creating bactericidal surfaces on various materials. Additionally, the microgel film’s cytocompatibility opens up opportunities for the biomedical field.

Koufakis et al. [105] prepared the poly(2-(dimethylamino)ethyl methacrylate) (PDMAEMA) quaternized brushes with quaternary ammonium groups of various alkyl chain lengths (ACLs) through a post-polymerization quaternization reaction. The PQDMAEMA brushes which were quaternized using short ACLs (C1-C3) displayed hydrophilic properties, attributed to the persistent positive charges existing on the sidal groups of the polymer. On the other hand, PQDMAEMA brushes with long ACLs (C ≥ 6) resulted in surfaces characterized as hydrophobic, showing the strong influence of alkyl chains on the films’ surface wettability. The alkyl chain length (ACL) of the quaternary ammonium salt moieties proved to be crucial in determining the bactericidal activity of the polymer brushes. Short ACLs resulted in hydrophilic, cationic, bactericidal brushes effective against Gram-positive and Gram-negative types of bacteria. On the other hand, ACLs with more than six carbon atoms led to non-bactericidal, hydrophobic and cationic surfaces. These findings offer valuable insights for designing effective cationic bactericidal polymer surfaces to combat biofilm formation.

Laube et al. [106] developed polymeric coatings composed of vinyl benzyl monomer units with quaternary ammonium groups, which were evaluated for their antimicrobial effectiveness on titanium surfaces. The QAC-containing monomers were polymerized directly on plasma-pre-activated titanium substrates using ATRP surface polymerization. For comparison, a conventional approach was also used, involving the copolymerization of QAC-containing monomers with a vinylbenzyl phosphonate monomer that adheres to the substrate, followed by applying the resulting copolymers to the titanium substrates through a drop-coating method. Variations in antibacterial activity were observed when altering the alkyl chain length of the QAC. In our experiments using *E. coli* as the test organism, a QAC with an octyl (C8) chain exhibited higher antibacterial activity compared to a QAC with an octadecyl (C18) chain.

In a study by Kalelkar et al. [107] brominated PLA surfaces were used as initiators for surface-initiated atom-transfer radical polymerization (SI-ATRP) of 2-(methacryloyloxy)ethyl]trimethylammonium chloride, a quaternary ammonium methacrylate (QMA). Grafting poly(QMA) brushes onto PLA films made them hydrophilic and resulted in a three-order of magnitude increase in antimicrobial effectiveness against Gram-negative bacteria, such as *Escherichia coli*, compared to unmodified PLA. This two-step approach to PLA surface modification offers a valuable method for developing PLA materials suited to biomedical and antimicrobial packaging applications.

Yu et al. [108] developed a thick poly((2-dimethylamino)ethyl methacrylate) (PDMAEMA) layer grafted onto PDMS using subsurface-initiated atom transfer radical polymerization (SSI-ATRP). Leveraging the tertiary amines in PDMAEMA, a one-step process enabled simultaneous zwitterionization and quaternization of the thick PDMAEMA layer. The results indicated that a high zwitterionization degree (75 mol%) combined with a low quaternization degree (25 mol%) provided an optimal balance between fouling resistance and bactericidal activity.

### 4.3. Dual-Functional Coatings

Bacterial adhesion and the subsequent formation of biofilms have been identified as the primary causes of bacterial-related infections and diseases [109]. Given the challenges mentioned above, there has been an increasing focus on creating multifunctional antibacterial coatings. These coatings combine bactericidal and anti-adhesion capabilities in a synergistic manner, resulting in significantly improved antibacterial efficiency through their combined action mechanisms. This is accomplished by introducing functional elements, such as QACs, onto anti-adhesion surfaces, thereby granting the surfaces bactericidal properties. This category includes coatings that are explicitly engineered to serve both purposes in a single material. The primary purpose of these coatings is now twofold: to repel bacteria and kill them upon contact.

Ye et al. [110] introduced a simple and universal approach to effectively combine superhydrophobic and antibacterial properties on different textiles. This method involves a one-step dip-coating or spray-coating process, where polydimethylsiloxane (PDMS) is utilized as a binder to attach fluorinated mesoporous silica nanoparticles (F-MSNs) as well as quaternary ammonium-functionalized MSNs (Q-MSNs) onto different textile surfaces. Notably, this technique does not require any specific pre-treatment or post-treatment steps. The newly created coating displayed important antibacterial efficiency against *E. coli* and *S. aureus* owed to the “repel-and-kill” synergistic action but also outstanding waterproofing and bacterial shielding properties.

Chen et al. [111] described an efficient dual-functional coating utilizing synthetic terpolymers, which combined dopamine, zwitterionic 2-methacryloyloxyethyl phosphorylcholine (MPC) and maleopimaric acid quaternary ammonium cation (MPA^−^N^+^). Random terpolymers were synthesized, comprising monomers where the biocidal component was maleopimaric acid quaternary ammonium cation (g-MPA^−^N^+^), the antifouling component was zwitterionic 2-methacryloyloxyethyl phosphorylcholine (MPC) and the anchoring moieties were dopamine methacrylamide (DMA). This dual-functional coating demonstrated significant therapeutic potential for addressing challenging clinical issues arising from pathogenic biofilms and the associated inflammation.

In another work, polyelectrolyte complex nanoparticles (PEC NPs) with adjustable quaternary ammonium groups using dextran sulfate sodium and chitosan (CS) were developed [112]. These nanoparticles serve as innovative building blocks to create quaternized polyelectrolyte complex membranes (QPECMs) for nanofiltration applications. The preparation method involves surface coating and glutaraldehyde crosslinking of the PEC NPs.

The study of Zhang et al. [113] presented an economical and environmentally friendly finishing method for cotton fabric surfaces, offering them antifouling as well as bactericidal properties. The approach combines the “repel-and-kill” strategies using two antimicrobial finishing agents: zwitterionic sulfopropylbetaine with isocyanate group (ISB) and quaternary ammonium salt with isocyanate group (IQAS). The coating of these agents onto the fabric is achieved through a simple dipping-padding-drying process. The zwitterionic sulfobetaine forms a bound surface on the fabric, effectively reducing initial bacterial attachment and delaying microbe colonization, resulting in a prevention of the formation of biofilms on the fabric surface. Meanwhile, the bound quaternary ammonium salt exhibits rapid and potent bactericidal activity against invasive and contacted pathogenic microbes, providing a long-lasting effect. In summary, this method offers a highly efficient way to prepare cotton fabric surfaces with antifouling and bactericidal functionalities, utilizing synergistic “repel-and-kill” mechanisms to achieve durable and effective antimicrobial properties.

In their research, Dai et al. [114], developed an antibacterial surface incorporating quaternary ammonium lactone rings and NCO groups. This surface exhibits the ability to kill bacteria and also demonstrates anti-bacterial adhesion and bacteria-releasing functions under alkaline conditions.

Moreover, a self-renewing and environmentally friendly coating was created by combining a self-polishing polymer (SP) with SiO_2_ nanoparticles tethered with quaternary ammonium salt (QAS) [115]. The synergistic effect of the biocidal QAS and hydrolysable SP under weak alkaline conditions resulted in an enhanced anti-biofouling property in the final coating.

Our research group has focused on improving biocidal activity and longevity by developing dual-action biocidal coatings. These materials simultaneously exhibit both contact killing and release-based biocidal actions. The contact killing activity typically relies on quaternary ammonium groups integrated through covalent bonding into a polymeric chain or substrate, while the release-mediated antimicrobial effect is achieved by incorporating biocidal agents able to leach from the polymeric surfaces or materials. To achieve this goal, our research group has synthesized newly designed random copolymers with reactive groups likecarboxyl or epoxide groups, incorporating quaternary ammonium antimicrobial groups in three different modes: contact-based action, release-based action and the combination of both. For the released-based polymeric biocidal materials, we have examined biocidal agents with 16-carbon atoms hexadecyltrimethylammonium (cetyl trimethylammonium) cations (AmC_16_) [116] and 12-carbon atoms dodecyltrimethylammonium bromide (DTAB) [117] electrostatically bound to an anionic poly(styrene sulfonate) (PSS) backbone. To achieve the contact-based action, we have developed polymers of 4-vinylbenzyl chloride (VBC) modified with biocidal agents with 16-carbon atoms N,N-dimethylhexadecylamine (HAM) [118], 3-carbon atoms trimethylamine (TEAM) [119] or 12-carbon atoms N,N-dimethyldodecylamine (DDA) [117]. Furthermore, for the combined actions, we have designed random or block copolymers of the corresponding units, hexadecyltrimethylammonium styrene sulfonate (SSAmC_16_) and 4-vinylbenzyl dimethylhexadecylammonium chloride (VBCHAM) [120]. Our research has demonstrated the successful application of the concept of reactive blending to create self-standing cross-linked membranes that incorporate released-based hexadecyltrimethylammonium groups and contact-based VBCHAM biocidal units [121]. First, the polymeric precursors of two biocidal species with complementary chemical functionalities, such as carboxylic groups from acrylic acid (AA) and epoxide groups from glycidyl methacrylate (GMA), were primarily synthesized. Subsequently, blends of P(SSAmC_16_-co-GMAx) and P(VBCHAM-co-AAx), were prepared and treated in the solid state at the required temperature, resulting in the formation of crosslinked membranes. This cross-linking occurred due to the carboxylic and epoxide group’s reaction. Notably, the above-mentioned membranes exhibited strong antimicrobial action targeting *S. aureus* and *P. aeruginosa*. In another work, we conducted a more comprehensive investigation of this methodology by varying the composition and blending ratio of different copolymers. The aim was to gain a more profound understanding of the structural factors that influence release behavior. This knowledge is crucial for optimizing the effectiveness and longevity of the antifouling action exhibited by these innovative polymeric biocidal coatings [122]. The cross-linking was also one factor thoroughly explored by testing various parameters, such as temperature, duration, mixing ratios of functional units, mixing portions of electrostatically and covalently attached biocidal groups [106], the cross-linking agents [123,124], to create cross-linked architectures suitable for coating different polymeric matrices, providing them with antimicrobial activity. Their application of aquaculture nets as coatings demonstrated significant antifouling properties in comparison to blank nets, either when used a single layer coatings [121] or as multi-layer coatings [125].

Aligned with this research, we have used selected pairs of complementary reactive copolymers to coat aquaculture nets for submerged marine applications. These coatings are designed to maintain their antifouling action for 35 days [121], which was extended to 66 days under scaled-up conditions [124].

In another work [126], copolymers incorporating quaternary ammonium (QA) and phosphorylcholine (PC) groups were successfully synthesized with the aid of free-radical copolymerization. The QA group imparts bactericidal properties, while the PC group mimics the cell membrane, offering antithrombotic and antifouling properties. To ensure long-term stability, a straightforward dip coating technique was employed to apply the copolymer coating. The presence of PC and cation groups led to surface inversion, transforming the coating from hydrophobic to hydrophilic. This inversion endowed the coatings with antithrombotic and antibiofilm properties. Over a week-long period, the coating exhibited sustained antibiofilm effects and reduced platelet adhesion and activation when compared to glass or PLA samples. Importantly, no hemolysis was observed for any of the coatings. The synthesized copolymer coatings with antithrombotic and antibiofilm properties hold great promise for their possible use in blood-contacting devices and implants, including heart valves, vascular stents and extracorporeal membrane oxygenation systems.

**Figure 7:**
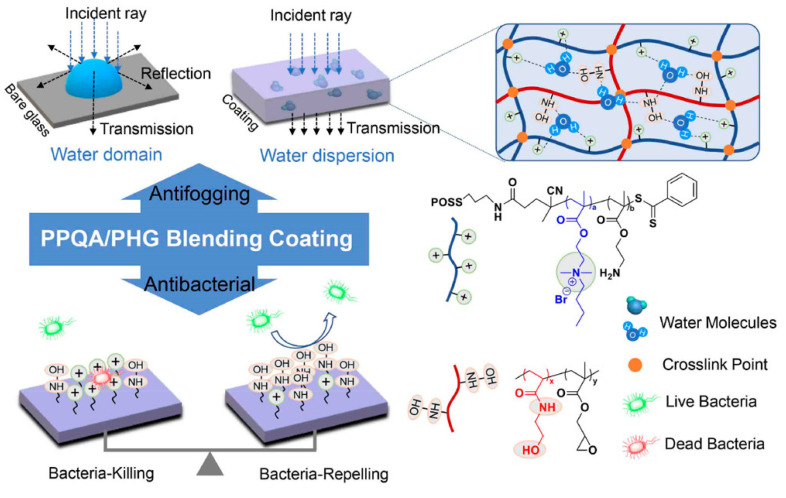
Antifogging and antibacterial performance of the PPQA/PHG blending coatings. The cationic as well as the hydroxyl-containing copolymers contribute to the antifogging performance through water absorption and diffusion, while also offering bacteria-repelling and killing properties [127]. Reprinted with permission from Bai et al. [127]. Copyright 2020 American Chemical Society.

Bai et al. [127] presented a novel approach to develop antifogging/antibacterial coatings by blending a cationic copolymer and a hydrophilic copolymer. The two copolymers employed were polyhedral oligomeric silsesquioxane-poly(quaternary ammonium compound-co-2-aminoethyl methacrylate hydrochloride) [POSS-P(QAC-co-AEMA)] and poly(N-hydroxyethylacrylamide-co-glycidyl methacrylate) [P(HEAA-co-GMA)]. The resulting transparent coatings exhibited outstanding antifogging performance in both in vitro and in vivo fogging conditions, which was primarily ascribed to the high capability of HEAA and QAC to absorb water. Additionally, due to the high hydratability of HEAA, the blended coatings demonstrated a bacteria-repelling behavior. The blending ratio adjustment between POSS-P(QAC-co-AEMA) and P(HEAA-co-GMA), led to coatings customization to achieve a comprehensive combination of bacteria-killing and bacteria-repelling properties (Figure 7).

Fu et al. [128] introduced a novel antimicrobial and antibiofouling coating with the use of a mixture of PHEAA and cationic PMETAC brushes, which exhibited remarkable antibacterial adhesion properties, maintaining an ultralow cell adhesion of approximately 3.0 × 10^5^ cells/cm^2^. They also demonstrated high contact killing efficiency, eradicating over 90% of attached *E. coli* and *S. aureus*. Additionally, the coating displayed prolonged antifouling activity even after 72 h of bacterial exposure. While the pure PHEAA brush showed excellent bacteria-repelling capabilities, it lacked bacteria-killing properties. Conversely, the pure PMETAC brush exhibited strong bacteria-killing abilities but had low resistance to bacterial adhesion. In contrast, the pure PMETAC brush exhibits potent bacteria-killing properties but shows limited resistance against bacterial adhesion. By combining the two brush types, the surface was endowed with both antimicrobial and anti-fouling functionalities.

As described by Wang et al. [129], a copolymer consisting of poly(2-(dimethylamino)ethyl methacrylate-co-2-methacryloyloxyethyl phosphorylcholine) (P(DMAEMA-co-MPC)) was grafted to the PDMS substrate using surface-initiated reversible-addition fragmentation chain transfer polymerization. Subsequently, quaternization was performed to provide the surface with dual capabilities for efficiently killing and repelling both Gram-positive and Gram-negative bacteria.

Khoerunnisa et al. [130] showed that when benzalkonium chloride (BAC) was incorporated as an antimicrobial agent, it enhanced the chitosan-based composite membranes’ toughness and durability, providing them with antibacterial effectiveness against *E. coli* and *S. aureus*. The gradual release of BAC from the novel composite membrane makes it an attractive candidate for preventing biofilm formation and biofouling.

### 4.4. Anti-Adhesion or Bacteria-Repelling Coatings

Anti-adhesive or bacteria-repelling coatings are designed to prevent microorganisms from attaching to the material at an early stage, ultimately halting the development of stable biofilms [131]. Despite the fact that significant progress has been made in the research on anti-adhesive or bacteria-repelling coating, there is a growing interest in the combination of this mechanism with others, such as contact killing for the development of antibacterial coatings with more favorable antibacterial effects. Thus, some coatings may exhibit both anti-adhesion and bactericidal properties. Certain coatings primarily designed for anti-adhesion (Section 4.4) may also demonstrate qualities relevant to this dual functionality (Section 4.3). This clarification highlights that the categories are organized based on each coating’s primary intended function, rather than on exclusive characteristics. Among the various design strategies that have been designed for the development of antibacterial surfaces in biomedical applications, quaternary ammonium coatings offer a flexible, safe and challenging concept. This is mainly due to the easiness of the processing and functionalization of quaternary ammonium salts, their antibacterial activity and a series of other favorable characteristics that they exhibit. This section focuses on coatings designed primarily to repel bacteria or prevent bacterial adhesion, even if they incidentally have some bactericidal effect. These coatings may not actively kill bacteria, but create surfaces that bacteria cannot easily attach to or colonize. The primary function is mainly anti-adhesion or bacteria-repelling.

Montefusco-Pereira et al. [132] developed innovative bacteria disruptors with reduced cytotoxicity and anti-adhesive properties by modifying of PEG-liposomes surface with linkers containing quaternary ammonium compounds (QACs) through the attachment of QAC (11-mercaptoundecyl)-N,N,N-trimethylammonium bromide (MTAB) to maleimide-functionalized liposomes (DSPE-PEG) via a thiol linker. The results showed that MTAB-functionalized liposomes effectively inhibit bacterial adherence and biofilm formation while also reducing the toxicity associated with MTAB.

According to a study by Qiu et al. [133], the poly(ether sulfone) ultrafiltration membrane was modified by incorporating the assembled composite of cetyltrimethylammonium bromide and MgAl-layered double hydroxide through the phase inversion technique. The resulting membrane demonstrated superior antibiofouling performance compared to the control PES membrane and effectively prevented biofilm formation even after undergoing several fouling–cleaning cycles.

Additionally, a successful modification of a polyvinylidene fluoride membrane was achieved using quaternized graphene oxide (QGO) [134]. The introduction of QGO indicated a great decrease of bacterial adhesion, an enhancement of membrane’s mechanical properties while also minimizing leaching into the environment, allowing it to keep antibacterial properties over an extended period.

He et al. [135] prepared dopamine-terminated quaternary ammonium salt polymer (D-PQAS) and dopamine-terminated PSBMA (D-PSBMA). These polymers were then effectively attached to the silicon substrate using a straightforward immersion technique inspired by the mussel adhesion method. As a result, the surface that was obtained had demonstrated a combination of anti-adhesive and bactericidal properties. The ratio of D-PSBMA to D-PQAS and the length of the N-alkyl chain of D-PQAS were found to influence the antibacterial performance of the modified surface.

Cao et al. [136] created an innovative protein-repellent and antibacterial dental resin based on polymethyl methacrylate (PMMA), incorporating 2-methacryloyloxyethyl phosphorylcholine (MPC) and quaternary ammonium dimethylaminohexadecyl methacrylate (DMAHDM). After 6 months of water-ageing, the dental resin containing PMMA-MPC-DMAHDM demonstrated higher flexural strength and elastic modulus compared to the PMMA control. The incorporation of MPC and DMAHDM in the novel PMMA resin resulted in powerful and long-lasting protein-repellent and antibacterial characteristics.

In a study by Kula et al. [68] a group of nine monomeric QASs, varying in aliphatic chain length (C12, C14 and C16) and counterion type (methylcarbonate, acetate and bromide), were evaluated for anti-adhesion properties on stainless steel, polystyrene, silicone and glass surfaces. The results indicated that compounds with 16-carbon hydrophobic chains were the most effective against both planktonic cells and biofilms. While all tested surfactants (C12, C14 and C16) demonstrated anti-adhesion activity, their effectiveness depended on the specific surface and bacterial strain used.

Zhang et al. [137] reported a slippery liquid-infused porous surface (SLIPS) with super-repellent properties. Using a simple microphase separation technique with poly(ethylene glycol) (PEG) as a sacrificial template, a polystyrene-based porous structure was created, modified with QAC-silane and infused with silicone oil to produce QAC-grafted SLIPS. Upon introducing silicone oil as a lubricant into the porous structure, the SLIPS surface exhibited exceptionally high super-repellence against both Gram-positive and Gram-negative bacteria. Additionally, it retained crucial contact killing antimicrobial activity from the fixed QAC-11 groups, even after the infused lubricant was depleted.

Mazurkiewicz et al. [89] prepared gemini quaternary ammonium salts (QAS) with various counterions, chain lengths (C12, C14 and C16) and a linker with three CH_2_ groups and tested their anti-adhesive, anti-biofilm and fungicidal action. The tested gemini QASs were evaluated for their effectiveness in reducing the adhesion of *C. albicans* cells to surfaces like glass, silicone, polystyrene and stainless steel. The results showed that these surfactants could successfully prevent adhesion of this strain to glass, silicone and stainless-steel surfaces with the most significant effects observed for compounds containing methylcarbonate, acetate and lactate counterions, particularly on silicone surfaces.

### 4.5. Intelligent Coatings

In recent years, substantial progress has been made in stimuli-responsive materials, which can be attributed to the combination of innovative synthesis methods and well-defined copolymer self-assembly [138]. The advancement of intelligent antimicrobial coatings addresses issues such as uncontrolled release of antimicrobial agents and residues of dead bacteria. Moreover, they significantly enhance the effectiveness of functional antimicrobial coatings [139,140]. These coatings offer outstanding antibacterial capabilities through the combined action of multiple antibacterial mechanisms and may achieve controlled release of antimicrobial agents in response to physical and chemical stimuli, thereby reducing environmental contamination caused by these agents and facilitating the removal of dead bacteria, extending the duration of antibacterial action. They have the potential to revolutionize the field of antibacterial protection, providing innovative and versatile solutions for addressing bacterial challenges more effectively while promoting better health and safety outcomes. Overall, these features make them currently the ideal choice for antimicrobial coating materials.

**Figure 8:**
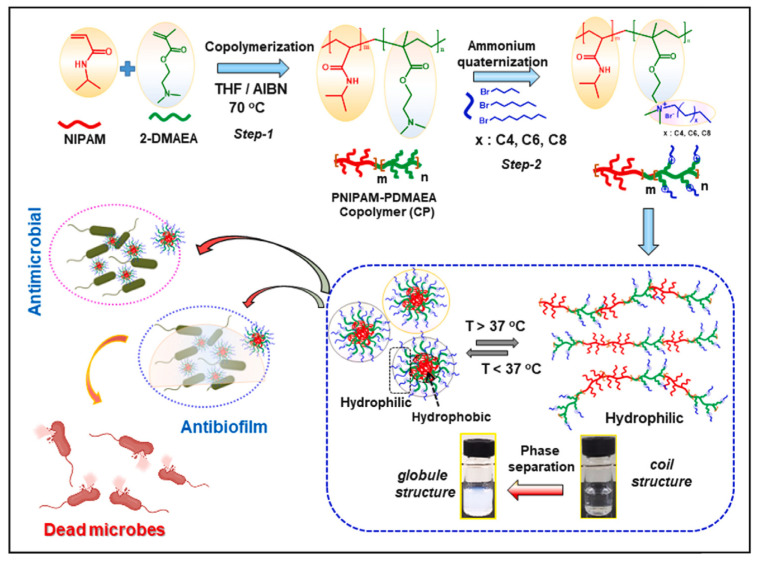
Copolymer (CP) and quaternary ammonium functionalized CP@QAS-Cn synthetic route and its antimicrobial and antibiofilm efficiency [141]. Reproduced with permission from Elsevier.

Raorane et al. [141] presented a novel synthetic method for the production of antimicrobial copolymers based on QAS with possible antibiofilm efficacy that may be applied in biomedical uses, as shown in Figure 8. More specifically, a novel copolymer with double functionality was successfully prepared using a one-pot copolymerization technique. In this process, N-isopropylacrylamide (NIPAM) and 2-dimethylaminoethylmethacrylate (DMAEA) monomers were copolymerized and then quaternized with alkyl bromide. As a result, thermoresponsive poly(N-isopropylacrylamide) (PNIPAM) segments were obtained. These PNIPAM segments displayed highly effective antimicrobial and antibiofilm properties, combining the attributes of quaternary ammonium salt (QAS)-based PDMAEMA segments within a single copolymer system. The study findings demonstrated that a shorter alkyl carbon chain length led to an improved antimicrobial effect. This enhancement was attributed to the existence of positively charged QAS groups, which were exposed on the outer layer of the micelles at higher temperatures, facilitated by the formation of a globular structure for the CP@QAS-Cn polymer. The dual functions of the thermoresponsive copolymer with quaternized segments, CP@QAS-Cn, such as thermoresponsiveness and antimicrobial efficacy, make it suitable for potential biomedical applications.

Zhan et al. [142] developed novel surfaces capable of switching their bioactivity in response to sugar. These surfaces rely on active covalent bonding among PBA-containing polymer brushes and functional CD-X molecules (X = lysine and a quaternary ammonium salt [QAS]). The bioactivity of these surfaces can be activated by attaching CD-X molecules combined with specific ligands under mild conditions in an aqueous solution and at room temperature. Conversely, the bioactivity can be deactivated by a simple treatment with a sugar solution, causing the release of CD-X from the surface. This system possesses remarkable generality and versatility, making it a promising biomolecule-responsive approach for designing dynamic bioactive surfaces. Its potential applications in the biomedical and biotechnology fields are extensive and diverse.

A covalently attached thermoresponsive antibacterial hydrogel layer was created on the surface of a polyether sulfone (PES) membrane by Wang et al. [143]. The hydrogel thin films were successfully synthesized through a photoinitiated copolymerization of NIPAAm, a thermoresponsive component, and DMC, a bactericidal component. Above the LCST (lower critical solution temperature), the surface became hydrophobic, effectively capturing live bacteria, and the hydrogel collapsed, exposing the bactericidal quaternary ammonium salt DMC to efficiently destroy the adhered bacteria. The surface turned hydrophilic, below the LCST, causing the hydrogel to swell, which rapidly reduced the adhesion force between membrane and bacteria. As a result, the dead bacteria detached, achieving a self-cleaning effect on the surface. The modified membranes demonstrated enhanced blood compatibility, as evidenced by prolonged APTTs (activated partial thromboplastin time) and no occurrence of hemolysis.

**Figure 9:**
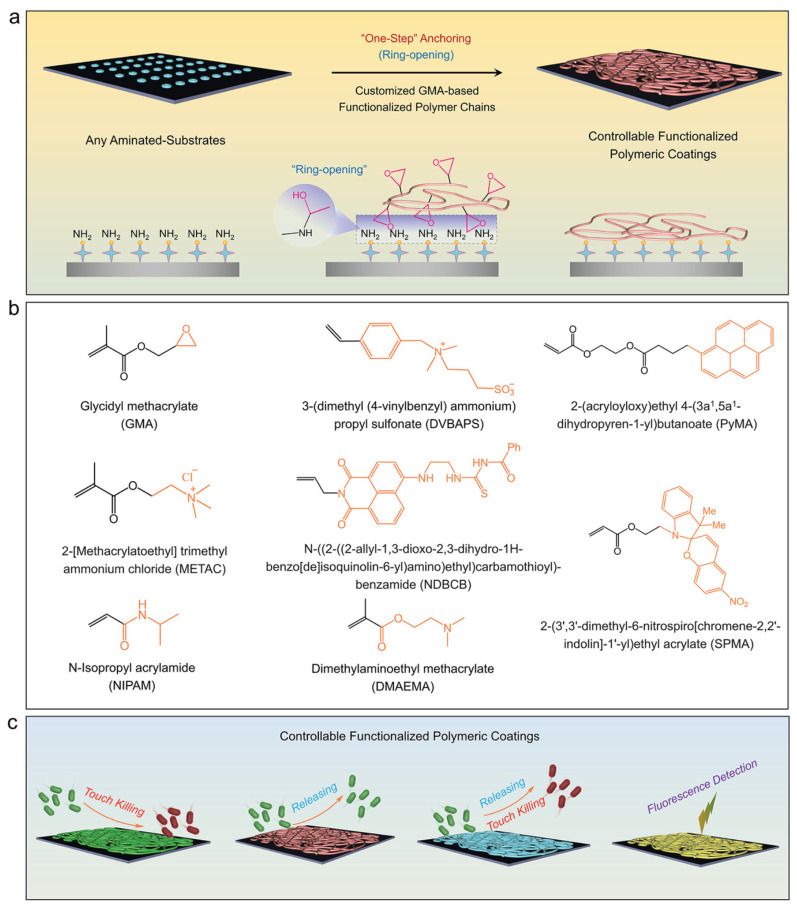
(**a**) A single-step, versatile surface coating method for anchoring various glycidyl methacrylate (GMA)-based, stimuli-responsive copolymers onto aminated substrates through a ring-opening reaction between the epoxy groups of GMA and the amine groups of the substrates. (**b**) GMA and seven stimuli-responsive monomers were copolymerized to create various surface adhesive and functional polymer coatings, including salt-responsive 3-(dimethyl (4-vinylbenzyl) ammonium) propyl sulfonate (DVBAPS), pH-responsive diethylaminoethyl methacrylate (DMAEMA), temperature-responsive N-isopropylacrylamide (NIPAM), antimicrobial 2-(methacryloyloxy) ethyl] trimethylammonium chloride (METAC), and fluorescent 2-(acryloyloxy)ethyl 4-(3a^1^,5a^1^-dihydropyren-1-yl)butanoate (PyMA), N-((2-((2-allyl-1,3-dioxo-2,3-dihydro-1H-benzo[de]isoquinolin-6-yl)amino)ethyl)-carbamothio-yl)-benzamide (NDBCB) and 2-(3′,3′-dimethyl-6-nitrospiro[chromene-2,2′-indolin]-1′-yl)ethyl acrylate (SPMA). (**c**) Copolymer coatings based on GMA were designed to perform distinct functions, including surface resistance to live bacteria, the release of dead bacteria, bacterial killing and the release and detection of heavy metal ions on the surface (Reproduced from Ref. [144], with the permission of Wiley).

Mao et al. [144] introduced a versatile and uncomplicated coating technique for achieving strong and complete connection of different stimuli-responsive GMA-based copolymers on a diverse array of substrates via a simple one-step ring-opening reaction, as shown in Figure 9.

This proposed method facilitates a strong interfacial addition of several stimuli-responsive polymers, such as temperature-responsive poly(GMA-co-NIPAM), pH-responsive poly(GMA-co-DMAEMA) and salt-responsive poly(GMA-co-DVBAPS), with surface adhesion epoxy groups in GMA. It can be applied to a wide range of hydrophobic and hydrophilic aminated solid substrates, comprising silicon, polypropylene, polyvinyl chloride, indium tin oxide (ITO), polyethylene terephthalate, polyvinylidene fluoride, aluminum, glass and polydimethylsiloxane. The resulting GMA-based copolymer coatings strongly attached to the substrates, exhibit impressive performance with distinct stimuli-responsive functionalities. These include a bacterial killing efficacy exceeding >95%, a thermal-, pH- and salt-responsive bacterial releasing efficacy of approximately 96%, a tunable fluorescence emission, and the ability to detect hypertoxic Hg^2+^ ions in an ion-responsive manner. In conclusion, this innovative membrane modification holds significant potential for widespread applications, imparting temperature-triggered, switchable bactericidal and antifouling properties to various surfaces.

**Figure 10:**
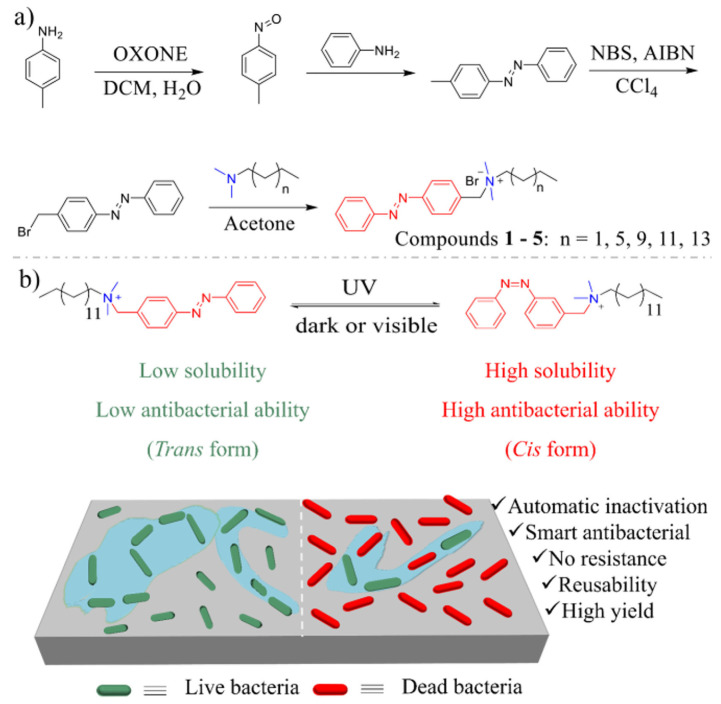
(**a**) Synthesis of smart antibacterial azobenzene-quaternary ammonium compounds **1**–**5** and (**b**) cartoon characterization of photosensitive antibacterial compound **4** [145]. Reproduced with permission from Elsevier.

A range of smart antibacterial photosensitive azobenzene-quaternary ammonium salt agents were synthesized by linking azobenzene with amines of varying chain lengths by Zhu et al. [145], as shown in Figure 10.

The goal was to enhance the quaternary ammonium salt’s (QAS) antibacterial selectivity and to prevent the active QAS accumulation in the environment. The trans-cis isomerization process resulted in increased solubility and enhanced antibacterial properties of the title compound (compound **4**). The results demonstrated that after 365 nm light irradiation, the antibacterial effect of compound **4** was notably augmented, showcasing its photosensitive intelligent antibacterial activity. Furthermore, the compound exhibited the ability to be reused for subsequent antibacterial applications.

Pang et al. [146] presented a zwitterionic, single layer polymer brush structured surface with charge-reversal capability in response to pH changes. The surface was created by grafting polydimethylaminoethyl methacrylate (PDMAEMA) chains onto a glass substrate. Further modification with phthalaldehydic acid (PADA) resulted in a smart antibacterial surface functionalized with zwitterionic polymers (G^−^N^+^–COO^−^) that could lower the adhesion of bacteria in a normal environment and then kill bacteria in an acidic environment, making it highly promising for use in implantable medical devices.

Our group developed novel ionic liquids-functionalized copolymers with double action: humidity responsiveness and antimicrobial activity [147]. Initially, random copolymers, P(VBCImCn-co-AA20), were synthesized composed of poly(4-vinylbenzyl N-alkyl imidazolium chloride-co-acrylic acid), with varying aliphatic chain lengths (n = 1, 4, 8, 12, 16 carbon atoms) for investigating how hydrophobicity/hydrophilicity impacts on the humidity-responsive properties of the copolymer as well as on its antimicrobial activity. Subsequently, blending of these copolymers with complementary reactive copolymers, of poly(cetyl trimethylammonium 4-styrene sulfonate-*co*-glycidyl methacrylate), P(SSAmC_16_-co-GMA20), followed in order to create stable films and coatings via thermal cross-linking. Among these blends, the membrane P(VBCImC_12_-co-AA20)/P(SSAmC_16_-co-GMA20), with a molar ratio of 3:1 (mol AA/mol GMA), demonstrated an immediate and pronounced response to moisture, folding or flipping after positioned on wet filter paper or in the palm of a hand. The inhibitory effects on the growth of specific bacterial species (*Escherichia coli*, *Pseudomonas aeruginosa* and *Staphylococcus aureus*) on the copolymer membranes depended on the length of the imidazolium alkyl chain and the particular bacterial species. The resulting stable, self-standing, and humidity-responsive monolayer membranes, along with their strong antimicrobial effectiveness, position the systems as promising contenders for real-world applications. These applications include sensors’ development in biomedicine and smart devices’ creation for controlled therapy and diagnostics.

## 5. Environmental Factors Affecting QAC-Based Antimicrobial Coatings

The performance of quaternary ammonium compounds-based antimicrobial coatings is largely affected by environmental factors like pH, temperature and humidity. Understanding these effects is crucial for the optimization of their efficacy and durability. These factors are analyzed in the following subsections.

### 5.1. pH Effects

The structural properties of QAC-based coatings and their antimicrobial efficacy can change drastically with variations in pH. For instance, acid conditions can increase QACs protonation and, thereby, the interaction of QACs with negatively charged bacterial membranes, enhancing their efficacy against microbes [148]. In contrast, for higher pH values, reduced protonation could result in poor antimicrobial activity. This is particularly relevant for biological environments, where pH may change due to metabolic activity of microorganisms [149]. Besides, the stability of the coating itself may be poor at extreme pH values, which negatively affects the release of the active antimicrobial agent. As pointed out by Xu et al. [150], interestingly a transition occurs at a pH of around 6.3 in the case of polymer brushes such as poly(2-(dimethylamino)ethyl methacrylate) (PDPA), which alters the antimicrobial and fouling-resistant property of these surfaces. This pH sensitivity will allow coatings to respond to the local environment, which is highly useful in the prevention of biofouling on medical devices. Cui et al. [151] further demonstrated that pH-responsive coatings, typically those made of polycaprolactone-copper peroxide composites, were more effective against bacteria at low pH. This was evidenced by the fact that such coatings attained a killing efficiency of over 99.99% against common pathogens like *E. coli* and *S. aureus* at pH 5.5. Therefore, these findings imply that the surrounding pH conditions can be designed in such a way that they could optimize the effectiveness of the action of QACs against microbes.

### 5.2. Temperature Effects

Temperature variations are another critical determinant in the effectiveness of antimicrobial coatings bearing QACs. Delbeke et al. [152] showed that temperature can influence the self-assembly properties of QACs, thereby affecting their antimicrobial activity. They reported that at higher temperatures, the nature of such amphiphilic compounds tends to disrupt the bacterial membranes much more effectively, enhancing their bactericidal action. Of course, some studies have indeed demonstrated that QAC-based coatings reveal their microbial action even at low temperatures, such as those relating to food preservation applications [153]. However, high temperatures may accelerate the degradation of the coating material, which may further lead to the release of harmful byproducts or to a reduction in the effectively active concentration of the active anti-microbial agent [154]. Thermal stability of antimicrobial coatings is essential for their application in environments that may experience temperature variations, such as surgical settings or during storage [155].

### 5.3. Humidity

Effects: Another vital factor that might influence the properties of antimicrobial coatings is humidity. In general, high humidity levels increase the release/leaching of QACs from the coating, which may initially increase the effectiveness of the latter in terms of antimicrobial activity, but later may contribute to various environmental risks and antibiotic resistance [156]. On the other hand, low humidity could also make coatings dry out, possibly reducing their effectiveness and leading to microbial growth [157]. Moreover, the effect of the humidity level on the physical properties of the coatings is an important feature with respect to mechanical integrity and surface adhesion. For example, highly hydrophilic coatings may absorb too much moisture, swell and even result in a certain delamination from a substrate [158].

## 6. Applications of QACs in the Biomedical Sector (Medical Implants, Dental Materials, etc.)

**Figure 11:**
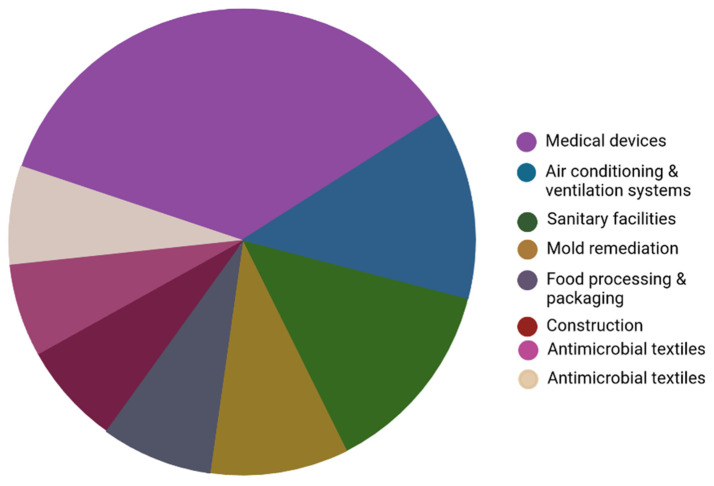
Applications of antibacterial coatings.

The demand for antimicrobial coatings has rapidly increased across various industries, including medical devices, ventilation systems and other industries, as shown in Figure 11. Biofilm formation presents a significant global health risk and causes substantial economic losses across multiple industries [159,160]. To combat biofilms effectively, it is crucial to focus on preventing the initial adhesion of microorganisms to substrates and their eradication even before the formation of the polymer matrix. This approach is recognized as the most effective strategy for combating biofilms. The design of antimicrobial coatings, materials and surfaces holds great promise as an effective approach to prevent biofilm formation [161]. In 2022, the worldwide market for antimicrobial coatings reached a total value of $10.12 billion (USD), and it is likely that a compound annual growth rate (CAGR) of 13.8% will be reached from 2023 to 2030. The increased emphasis on cleanliness across different professional sectors has driven the rapid growth of this industry. These coatings are intended for the protection of surfaces against a range of microorganisms, including parasites, germs, bacteria and other undesirable microorganisms [162].

Throughout the COVID-19 pandemic, the need for antimicrobial coatings experienced a substantial rise across diverse sectors, including electronic products, protective clothing and consumer goods. Notably, organizations like Droom, ZAGG Inc and others proactively adopted antimicrobial coatings for their products. In an effort to combat diseases, an IIT Madras start-up started the production of textiles coated with antimicrobial materials. The increasing recognition of hygiene’s significance is a key driver for the antimicrobial coatings market [163]. The market’s expansion can be attributed to several key factors, including the rising awareness regarding safety and hygiene, the implementation of stringent regulations, and the growing adoption of antimicrobial coatings across various industrial applications. Furthermore, in the medical field, antimicrobial coatings are favored by doctors over disinfectants and cleaning agents due to their ability to inhibit the adherence of germs to the surface of medical devices, thus effectively reducing the spread of infections [2].

Moreover, the use of antimicrobial coatings, such as silver-based coatings has raised concerns regarding health issues and ecotoxicological effects linked to silver usage, along with its substantial release (up to 100%) into the aquatic environment from antibacterial coatings, leading to their limited use [164,165]. Consequently, there is a significant interest within the material science community in exploring new antimicrobial agents that are both environmentally friendly and effective for surface modification.

Extensive research has been conducted on quaternary ammonium compounds (QACs) for the development of antimicrobial coatings, primarily due to their wide-spectrum bactericidal properties [166,167].

There is active investigation concentrated on exploring the use of QAC-based coatings in various applications, including wound dressings, dental materials, biomedical device modification, bone cements, effluent treatment, steel protection against corrosion and marine biofouling, among other areas. Gharibi et al. [168] used dextran, a polysaccharide that affects coagulation hemostasis, to compensate for the hostile impacts of quaternary ammonium salts (QAS) embedded in the structure of a wound dressing membrane that served as antimicrobial. The recorded findings validated that surface anchored dextran effectively regulated the hemocompatibility of QAS-containing wound dressings without compromising their fundamental physical and biological characteristics.

Quaternary ammonium compounds (QACs) have gained significant attention as potential contact killing antimicrobial materials for orthopedic implants. They are highly promising due to their broad antibacterial spectrum and low toxicity, making them suitable candidates for use in this field [169]. Abid et al. [170] successfully synthesized quaternary ammonium dendrimers using tripropylene glycol diacrylate (TPGDA) and confirmed their prospective utility as effective antimicrobial agents in bone cement. These dendrimers showed the ability to eliminate common hospital-derived bacteria, including *E. coli*, *S. aureus* and *P. aeruginosa*, through direct contact, maintaining sustained bactericidal activity for 30 days. Importantly, the modified bone cement displayed no significant cytotoxicity, as confirmed by the MTT assay. This suggests that TPGDA holds great promise for clinical use as an antimicrobial agent without compromising the mechanical properties of the cement.

Quaternary ammonium compounds (QACs) have also used been as potential contact killing antimicrobial materials for dental resins [171]. Dental adhesives containing benzyldimethyldodecylammonium chloride have also shown an impressive reduction in bacterial growth on biofilms formed on QAC-modified samples [46]. Furthermore, QACs’ addition to commercial giomers exhibit effective antibacterial properties showing significant potential in controlling the compositions of cariogenic biofilms [172]. Silva et al. [173] conducted a study to assess the cytocompatibility of keratinocytes and the ultimate tensile strength of dental resins containing myristyltrimethylammonium bromide (MYTAB). They examined different MYTAB concentrations and their effects on bacterial growth and mechanical properties. The results showed that a 2% MYTAB concentration reduced bacterial growth without compromising the tensile strength of the resins. However, a 1% MYTAB concentration significantly hindered cell viability to around 50%. Based on their findings, the researchers suggested that a concentration of 0.5% MYTAB would provide appropriate antibacterial action to the dental resins while maintaining adequate physical and chemical stability and a high level of biocompatibility, approximately 90%. Moreover, the incorporation of quaternary ammonium dimethylaminohexadecyl methacrylate into the dental resin structure showed strong antibacterial effectiveness for the resin with 3% DMAHDM against three streptococcal bacterial biofilms (*S. mutans*, *S. sanguinis*, *S. gordonii*), resulting in biofilm CFU reductions of 3–4 log, with consistent results observed from passage 1 to passage 20, as shown by Wang et al. [174]. Similarly, the incorporation of alkyl trimethylammonium bromide in orthodontic resins leads to reduced viability of both biofilm and planktonic forms of bacteria [175]. Li et al. [176] synthesized three bi-quaternary ammonium methacrylates (biQAMA-12, biQAMA-14 and biQAMA-16) with different alkyl chain length (12, 14 and 16), which were further incorporated into Bis-GMA/TEGDMA based dental resin composites (DRCs) as antibacterial agents. The addition of 5 wt% of biQAMAs to the resin matrix resulted in DRCs (Dental Restorative Composites) with over 90% antibacterial efficacy against *S. mutans*, while DRCs containing biQAMA-12 showed nearly 100% antibacterial activity. Incorporating 5 wt% of biQAMAs did not adversely affect the physicochemical properties of the DRC, except for an increase in water sorption. Among the biQAMAs tested, only biQAMA-12 did not influence the cytotoxicity of the DRC and was identified as the most effective antibacterial agent for DRC in this study. Jung et al. [177] utilized amphiphilic quaternary ammonium chitosan and sodium alginate to create multilayer films, aiming to inhibit fungal biofilm formation on denture biomaterials. These LbL (layer-by-layer) self-assembled coatings demonstrated excellent biocompatibility and stability, as confirmed by the absence of leaching in stability tests. A method combined surface grafting with electron beam irradiation was employed by Thongthai et al. [178] to enhance the resistance of dental resins against the formation of multispecies biofilms. The QAC-based monomer used in this process was 12-Methacryloyloxydodecylpyridinium bromide and the antibacterial and antibiofilm effectiveness was demonstrated against *Streptococcus mutans* as well as bacteria from human saliva.

Zhang et al. [179] developed a novel approach for creating antibacterial surface coatings with unique antifouling–bactericidal switching properties to combat catheter-induced infections (CAI). This strategy involves constructing convertible hierarchical polymer brushes through Surface-Initiated Atom Transfer Radical Polymerization (SI-ATRP) and the Schiff base reaction on the surface of polyurethane (PU). The coating referred to as PU-PQ-PEG comprises two distinct layers: poly[2-(dimethyl decyl ammonium)ethyl methacrylate] (PQDMAEMA) brushes, which is the bactericidal lower layer and polyethylene glycol (PEG), which is the antifouling upper layer. In typical and mild infection scenarios, PU-PQ-PEG exhibited outstanding antifouling properties, effectively resisting protein and bacterial adhesion while remaining biocompatible. However, during severe infections when bacteria was colonized on the PU-PQ-PEG surface, the coating displayed a remarkable self-adaptive antifouling–bactericidal switching mechanism, which was triggered by the presence of bacteria. In in vivo infection conditions, PU-PQ-PEG coatings maintained their antifouling behavior, resisting bacterial colonization under mild infection conditions. However, under severe infection conditions, the coatings effectively switched to a bactericidal mode, preventing bacterial growth. This adaptive nature of the surface coatings, combined with their biocompatibility, offers a promising approach for preventing catheter-induced infections. In a study by Song et al. [180], gelatin monolayers were coated on the surface of Ti with the use of an electrostatic method. For the enhancement of Ti implants’ surface biocompatibility as well as antibacterial properties and the improvement of their bone integration ability, quaternary ammonium bis epoxide salt (DEQAS) and quaternary ammonium Balearic acid salt (MPA^−^N^+^) were synthesized and grafted onto the collagen monolayer. The coated titanium implant resulting from this process exhibited remarkable antibacterial properties and significantly improved cell adhesion and migration. This material holds great potential for diverse applications in orthopedics, transplantation and surgery.

In a study by Xing et al. [181], a series of Ti specimens underwent surface-negative ionization with the use of a hot alkali activation technique. Subsequently, polylysine and polydopamine layers were placed on the surfaces using a layer-by-layer self-assembly approach. Following this, a quaternary ammonium salt (QAS) (EPTAC, DEQAS, MPA^−^N^+^) was grafted onto the coating. A total of 17 composite coatings were prepared using this method. The coated specimens demonstrated high bacteriostatic rates of 97.6 ± 2.0% and 98.4 ± 1.0% against *Escherichia coli* and *Staphylococcus aureus*, respectively. These results suggest that the composite coating holds promise in enhancing the osseointegration and antibacterial performance of implantable Ti devices.

Liu et al. [182] designed a novel antibacterial hydrogel through a straightforward and gentle process by the modification of a photocross-linkable gelatin-based polymer (GelMA) bearing cationic quaternary ammonium salt (QAS) groups. By controlling the hydrophobic carbon chain’s length on the QAS group and the extent of functionalization, the resulting GelMA-octylQAS hydrogel showcased a remarkable combination of desirable attributes. These included excellent mechanical properties, biodegradability, potent bactericidal activity against diverse types of bacteria and high compatibility with mammalian cells. When implemented on an implant using an in situ cross-linking method, our hydrogel exhibited exceptional antimicrobial abilities in a rat femoral fracture infective model. Based on these results, the GelMA-octylQAS hydrogel shows great potential as a platform for orthopedic and skin trauma surgery to prevent implant-associated infection challenges.

Jia et al. [183] introduced a novel approach for the hemocompatibility of QAC-polymer coatings, enhancing its potential application in blood-contacting devices and implants. They synthesized mixed-charge copolymers comprising poly(quaternized vinylpyridine-co-n-butyl methacrylate-co-methacrylic acid) through free radical copolymerization. The resulting copolymers were dissolved in a mixture of methanol and ethanol, combined and then applied onto glass slips using the ultrasonic spray technique. By incorporating an anionic group into the copolymer, the researchers significantly improved the coating’s hemocompatibility without losing its contact kill bactericidal properties.

## 7. Conclusions and Future Perspectives

Antibacterial coatings utilizing quaternary ammonium compounds (QACs) have proven to be highly effective in controlled release applications due to their low cost and accessibility, a fact that has become increasingly relevant following the COVID-19 outbreak. This research emphasizes the necessity for an in-depth understanding of the design considerations for QACs in the biomedical field. Our findings clarify the mechanisms by which these materials act and delineate the key structural and functional parameters that influence their antibacterial efficacy. We have also highlighted the various types of QAC-based antibacterial coatings and their specific uses in biomedical applications.

To achieve these objectives, we have identified several specific research directions and associated technical challenges that can guide future work on QAC-based antibacterial coatings:

Nontraditional applications (non-contact devices): While QAC-based coatings are widely used in direct-contact biomedical devices, their potential for non-contact medical devices (e.g., diagnostic sensors, medical imaging equipment and touchless interfaces) remains underexplored. Research should focus on adapting QAC coatings to these environments, where physical contact is minimal but antimicrobial protection is still crucial. Key questions include: How can QACs be applied to materials typically used in non-contact devices, such as glass, plastics or metals? What modifications are needed to ensure effective antimicrobial activity without compromising device performance or durability? Investigating these aspects could expand the scope of QAC coatings in infection control management beyond traditional medical applications.

Smart and responsive coatings: Integrating QACs with smart-release or responsive mechanisms could significantly enhance their versatility. For example, coatings that release QACs in response to environmental triggers (e.g., pathogen presence, pH shifts or temperature changes) offer potential for targeted antibacterial action. Specific research questions include: What environmental triggers are most suitable for activating QAC release in medical applications? How can we control the timing and rate of QAC release to ensure consistent, prolonged efficacy without premature depletion? Technical challenges involve designing responsive mechanisms compatible with QAC coatings and ensuring predictable activation in clinical settings.

Biocompatibility and sustainability: Developing biodegradable and non-toxic QAC coatings is vital for safer, more environmentally friendly biomedical applications. This includes exploring materials that maintain antimicrobial efficacy while being safe for both the patient and the environment. Relevant research questions include: What biocompatible materials can support the effective delivery of QACs without compromising antibacterial properties? How can we ensure that biodegradable QAC coatings are durable enough for medical applications without requiring frequent reapplication? Technical challenges here involve achieving a balance between biodegradability, safety and durability, requiring innovation in material science and formulation techniques.

Addressing bacterial resistance: Given the growing concern about bacterial resistance, it is crucial to understand how bacteria may adapt to QACs and to design coatings that minimize resistance. Key research questions include: What structural modifications to QACs could reduce the likelihood of bacterial resistance? How do specific bacterial strains respond to prolonged QAC exposure, and what coating designs can counter resistance mechanisms? Addressing these questions will involve studying bacterial adaptations to QACs in various settings and designing coatings that maintain efficacy over extended periods without encouraging resistance.

Clinical translation and regulatory approval: Moving QAC-based coatings from lab research to clinical application involves scaling up production and navigating regulatory requirements. Research questions include: What coating parameters (e.g., thickness and release rates) are necessary to meet regulatory standards for clinical-grade materials? How can production methods be adapted to create clinically viable, cost-effective QAC coatings? Technical challenges involve aligning lab-scale coatings with industry standards and ensuring cost-effective, reproducible production that complies with regulatory guidelines for safety and efficacy.

Future research should focus on developing dual-function antibacterial coatings, aiming to enhance their overall performance and expand their practical applications in the biomedical domain. Optimizing the structural parameters of quaternary ammonium compounds (QACs) is essential for improving their stability, release rates and overall antibacterial effectiveness. Advances in polymer chemistry and materials science could lead to the development of more effective and durable coatings. Additionally, integrating QAC-based coatings with advanced technologies, such as smart release systems or self-cleaning surfaces, has the potential to enhance their functionality. Research into incorporating sensors or triggers for on-demand release or activation is also promising.

Developing environmentally friendly and biocompatible QAC-based coatings is crucial for reducing environmental impact and ensuring safety in biomedical applications. Investigating biodegradable materials and non-toxic alternatives will be key for achieving sustainable development. Moreover, understanding how bacteria might develop resistance to QACs and designing coatings to counteract or prevent such resistance will be vital for ensuring long-term effectiveness. Finally, transitioning from laboratory research to clinical trials and securing regulatory approvals are necessary steps for making innovative QAC-based coatings practical and widely usable in biomedical settings. Addressing these future directions will significantly enhance the efficacy and application range of quaternary ammonium compound-based antibacterial coatings.
